# Patient Experience Drivers of Overall Satisfaction With Care in Cancer Patients: Evidence From Responders to the English Cancer Patient Experience Survey

**DOI:** 10.1177/2374373519889435

**Published:** 2019-11-25

**Authors:** Mayam Gomez-Cano, Georgios Lyratzopoulos, Gary A Abel

**Affiliations:** 1 171002 University of Exeter Medical School, University of Exeter, Exeter, United Kingdom; 2 Epidemiology of Cancer Healthcare & Outcomes (ECHO) Group, Department of Behavioural Science and Health, Institute of Epidemiology and Health Care, 4919 University College London, United Kingdom

**Keywords:** cancer, health-care planning or policy, patient satisfaction, survey data

## Abstract

**Background:**

Surveys collecting patient experience data often contain a large number of items covering a wide range of experiences. Knowing which areas to prioritize for improvements efforts can be difficult.

**Objective:**

To examine which aspects of care experience are the key drivers of overall satisfaction with cancer care.

**Methods:**

Secondary analysis of the National Cancer Patient Experience Survey. Logistic regression was used to examine the relationship between overall satisfaction and 10 core questions covering aspects of experience applicable to all patients. Supplementary analyses examined a further 16 questions applying only to patients in certain groups or on specific treatment pathways.

**Results:**

Of 68 340 included patients, 58 697 (86%) rated overall satisfaction highly (8 or more out of 10). The strongest predictors of overall satisfaction across all models were responses to 2 questions on experience of care administration and care coordination (odds ratio [OR] = 2.11, 95% confidence interval [95% CI = 2.05-2.17, *P* < .0001; OR = 2.03, 95% CI = 1.97-2.09, *P* < .0001, respectively, per 1 standard deviation change).

**Conclusion:**

Focusing improvement efforts on care administration and coordination has potential to improve overall satisfaction with oncological care across diverse patient groups/care pathways.

## Introduction

A positive experience of health care is increasingly considered a key dimension of care quality, alongside clinical quality and safety outcomes (1). Consequently, there is increasing investment in the systematic, nationwide, measurement of the quality of patient experience to guide improvement actions (2,3). Policy initiatives in this field initially did not focus on specific diseases but survey instruments specific to cancer patients have since been developed. In the United States, since 2016, questions capturing the experience of cancer patients can be incorporated into the CAHPS Clinician & Group Survey (CAPHS Cancer Care Survey) (4). In England, the experience of patients undergoing cancer care is measured by the Cancer Patient Experience Survey, which has been conducted in different waves since 2010 (5). Relatedly, the National Cancer Strategy in England indicates that patient experience should be considered “as being on a par with clinical effectiveness and safety” (6).

Questionnaires used in patient surveys typically encompass several aspects of care experience and many different questions. A common major challenge for clinicians and policy-makers using the findings of patient surveys is how to prioritize improvement efforts across the many different aspects of experience measured in such surveys. As patient surveys often include one or more general satisfaction items, one approach to this problem is to examine which aspects of care experience are most strongly associated with overall satisfaction (7,8). These key drivers of satisfaction are potential targets for quality improvement efforts which are aimed at addressing aspects of care experience that matter most to patients.

The (English) National Cancer Patient Experience Survey 2015 questionnaire includes many evaluative questions covering the experience of diagnosis, diagnostic testing, shared decision-making, specialist nursing, inpatient care, anticancer treatment (surgery, radiotherapy, chemotherapy), hospital discharge, and care in the community, together with an overall item for overall satisfaction with care. Cancer care typically involves multiple providers of care and multiple episodes of diagnostic testing/assessment and treatment sessions over time. Therefore, the survey instrument encompasses all key aspects of the cancer care pathway, from prediagnosis to use of investigations and certain treatment modalities (and hospital care) to discharge in the community. Survey results are reported publicly for each English hospital to support improvement efforts. Although a number of dimensions of care experience can be hypothesized as important, including provider communication skills, shared decision-making, access to and experience of specialist cancer nursing, and sense of holistic or integrative care, there is little evidence to suggest which of those matters most for patients (9–13). To best support prioritization of improvements on aspects of care experience that drive patient satisfaction, we aimed to identify which survey items are most strongly associated with overall satisfaction with cancer care.

## Methods

### Data

*Data source*: Data were analyzed from 71 186 respondents to the National Cancer Patient Experience Survey 2015 (response rate 65.7%). Details of the survey and method of administration have been published previously (14). Briefly, the survey was mailed to all adult patients (aged 16 and older) discharged from a National Health Service (NHS) hospital after inpatient or a day case cancer-related treatment during April to June, 2015 after vital status checks at survey mail-out (between 3 and 5 months after the sampling period). Cancer-related treatment was determined by the presence of primary diagnosis of cancer in hospital records (*International Classification of Diseases, 10th Revision* code (Fourth Edition) C00-C99 or D05, excluding nonmelanoma skin cancer, C44, and peripheral and cutaneous T-cell lymphomas, C84). Responses could be made by post, online, or using a telephone translation service. Information on patient age, gender, cancer diagnosis, and a measure of socioeconomic status based on hospital records was available with the anonymous data set, accessed via the United Kingdom Data Service (5).

*Care satisfaction (outcome) variable*: The National Cancer Patient Experience Survey 2015 includes a question about overall satisfaction with cancer care on a 0 to 10 scale (least-to-most satisfied, respectively) (5). Scores for this question had a very skewed distribution. We dichotomized responses such that a patient was considered satisfied if they scored 8 to 10 and less than satisfied if they scored 0 to 7. This binary version of the care satisfaction question was used as our outcome measure in all our models.

*Patient experience (exposure) variables*: The survey also includes 49 evaluative questions relating to key domains of patient experience (eg, interpersonal skills of care providers, the provision of information about the cancer diagnosis and its treatment, care access, care timeliness, care coordination, and anticipatory care) (5). These items comprise Likert scale response options with varying numbers of possible options. To enable comparison across these items, we scored response options for each question such that the scores were evenly spaced and had a mean of 0 and a standard deviation (SD) of 1 across all responders (in effect standardizing responses to a standard normal). This means that the resulting odds ratios [ORs] can be viewed as the effect of a 1 SD change on responses to a question.

Certain experience items did not apply to all patients (for example, questions on radiotherapy only applied to patients treated by this modality). For this reason, a complete case analysis including responses to all domains of care would be restricted to a very small atypical group of patients (eg, patients who had recently had a diagnostic test, an operation, and chemotherapy and radiotherapy, treated both as an in- and outpatients). Given this, we classified all questions into those that applied to nearly all patients and those which only applied to specific patient groups.

## Statistical Analysis

*Stage 1: Identifying the strongest predictors (of satisfaction) within domain with more than one question.* Certain questions related to the same experience domain as other questions. This poses a problem because including such questions as individual variables in regression models may result in the association being ”shared” between the questions, in effect diluting the perceived importance of the respective domain. Given this, the 49 evaluative questions were assigned to 26 domains of care, nested within the group of patients they applied to, independently by 2 authors (GAA and GL). Discrepancies were discussed and consensus reached including all authors. Individual domains contained up to 5 questions with 13 of the 26 domains containing only 1 question. A series of logistic regression models were used to predict overall satisfaction from all questions within a single domain (also adjusted for patient age, gender, deprivation, and tumor group, where deprivation is classified according to the quintiles of the Index of Multiple deprivation) (15). The strongest predictor of satisfaction within each domain was then retained for subsequent analysis.

*Stage 2: Main analysis.* Of the 26 retained questions, 10 core questions have been deemed applicable to nearly all patients. Even so, only 42% (29 669) of respondents answered all core questions. Multiple imputation by chained equations under the missing at random assumption was used to impute missing responses to core questions only, concordant with previous studies examining drivers of satisfaction in patient surveys (7,8). Moreover, we used predictive mean matching which maintains the interval nature of the data. Those questions represent domains of care that can be assumed to be relevant to all respondents. We did not impute responses to questions patients were not supposed to answer, as we considered it inappropriate to do so. Ten imputed data sets were created. We then fitted a logistic regression model including all core questions and patient variables for age, gender, deprivation, and tumor group. Model estimates were combined using Rubin’s rules (16).

*Supplementary analyses*: We aimed to examine whether the findings arising from the “core” model were applicable to different patient groups, for example, those with different care pathways. First, we ran a series of models where the core model was augmented by additional questions specific to certain patient groups/pathways, restricted to patients with domain-specific responses (a total of 12 supplementary models, one for each domain-specific patient group). Second, we reran the main analysis model restricted to the subsamples of patients who were included in each of the domain-specific models. These (12) models were restricted to each specific patient group/pathway, but were otherwise identical in structure to the main analysis model, that is, they did not include the items defining each specific patient group/pathway. The purpose of these models was to examine whether any resulting differences in the effect size of “core” questions between the main and supplementary analyses were due to confounding/mediation or sample restriction of the patient group/pathway-specific models. In other words, we aimed to distinguish whether aspects of care only experienced by a subgroup of patients in a specific patient group/pathway altered the strength of associations between overall satisfaction and common aspects of experience (eg, through a related mechanism) or whether these patients were inherently different in terms of what mattered to them. All analyses were conducted in STATA 15.

## Results

The demographic and clinical characteristics of respondents to the 2015 cancer patient experience survey are summarized in Table 1. Among 68 340 respondents who answered the question on overall satisfaction 58 697 (85.9%) rated it with scores of 8 or more out of 10. Male patients, those aged 65 to 74 and older deprived patients were more likely to be satisfied with their care, as were patients with testicular cancer, ductal carcinoma in situ, and leukemia.

**Table 1.** Demographic and Clinical Characteristics of Respondents to the 2015 CPES survey.

		N (% of Sample)	N Satisfied(% of Group)
Tumor group	Anal	282 (0.4%)	226 (80.1%)
	Bladder	4838 (6.8%)	3835 (79.3%)
	Breast	13 673 (19.2%)	11533 (84.3%)
	Cervical	332 (0.5%)	279 (84%)
	Colon	4896 (6.9%)	4018 (82.1%)
	DCIS	889 (1.2%)	774 (87.1%)
	Endometrial	1499 (2.1%)	1253 (83.6%)
	Leukemia	2564 (3.6%)	2229 (87%)
	Lip oral cavity and pharynx	1316 (1.8%)	1104 (83.9%)
	Lung	3693 (5.2%)	2980 (80.7%)
	Melanoma	1744 (2.4%)	1511 (86.7%)
	Multiple myeloma	4948 (7%)	4148 (83.8%)
	Non-Hodgkin lymphoma	4752 (6.7%)	4108 (86.4%)
	Oesophageal	1316 (1.8%)	1088 (82.7%)
	Other	4571 (6.4%)	3658 (80%)
	Ovarian	1821 (2.6%)	1495 (82.1%)
	Pancreatic	654 (0.9%)	505 (77.2%)
	Prostate	6168 (8.7%)	4981 (80.8%)
	Rectal	3312 (4.7%)	2733 (82.5%)
	Renal	992 (1.4%)	754 (76%)
	Secondary	4742 (6.7%)	3745 (79%)
	Soft-tissue sarcoma	570 (0.8%)	480 (84.2%)
	Stomach	864 (1.2%)	677 (78.3%)
	Testicular	221 (0.3%)	195 (88.2%)
	Thyroid	529 (0.7%)	388 (73.3%)
	Missing	0 (0%)	
Gender	Male	32 873 (46.2%)	27 267 (82.9%)
	Female	38 313 (53.8%)	31 430 (82%)
	Missing	0 (0%)	
IMD quintile	1	9254 (13%)	7367 (79.6%)
	2	12 306 (17.3%)	9895 (80.4%)
	3	15 547 (21.8%)	12 852 (82.7%)
	4	16 592 (23.3%)	13 901 (83.8%)
	5	17 076 (24%)	14 345 (84%)
	Missing	411 (0.6%)	
Age group	<55	10 669 (15%)	8499 (79.7%)
	55-64	14 267 (20%)	11 667 (81.8%)
	65-74	24 926 (35%)	21 024 (84.3%)
	75+	21 324 (30%)	17 507 (82.1%)
	Missing	0 (0%)	

Abbreviations: CPES, Cancer Patient Experience Survey; DCIS, ductal carcinoma in situ; IMD, Index of Multiple deprivation.

^a^ N = 71 186.

The questions identified as the strongest predictors of satisfaction within domains applying to all patients are shown in Table 2 along with the results of the regression analysis for the core model examining these questions. The question on care administration (Q56) was the strongest predictor of overall satisfaction (OR per 1 SD change = 2.11, 95% confidence interval [95%CI] = 2.05-2.17, *P* < .0001) followed closely by the question on coordination of care (Q54; OR per 1 SD change = 2.03, 95% CI = 1.97-2.09, *P* < .0001). The question on shared decision-making about treatment (Q16) and the question on relatives being given enough information to care for patients at home (Q49) were the next strongest predictors, although associated ORs were substantially weaker than those for the questions regarding care administration and care coordination.

**Table 2.** Results of Core Model Predicting Overall Satisfaction.^a^

Number of Observations	Question Number	Synoptic Form of Question	OR^b^	LCI^b^	UCI^b^
67 953	2	Length of time to wait before first appointment at hospital	1.20	1.17	1.23
	11	Written information about diagnosis	1.12	1.09	1.15
	16	Involved in the decisions about care and treatment	1.44	1.40	1.48
	17	Given the name of a CNS	1.10	1.07	1.12
	49	Relatives had all info the help care for you at home	1.38	1.33	1.43
	52	GP had enough info	1.05	1.02	1.07
	54	People treating and caring work well together	2.03	1.97	2.09
	55	Have you been given a care plan	0.98	0.94	1.02
	56	Overall, how would you rate the administration of your care	2.11	2.05	2.17
	58	Asked about taking part in cancer research	1.05	1.02	1.08

Abbreviations: CI, confidence interval; CNS, central nervous system; GP, general practitioner; LCI, lower confidence interval; OR, odds ratio; SD, standard deviation; UCI, upper confidence interval.

^a^ Also adjusted for patient age, gender, deprivation, and tumor group.

^b^ Odds ratios and 95% CI correspond to a 1 SD change in question response.

The only core question that was not a statistically significant predictor of overall satisfaction was the question on having been given a care plan (Q55).

### Supplementary Analyses

The questions identified as the strongest predictors of satisfaction within domains applying to specific patient groups/pathways are shown in Table 3 along with the regression results for these noncore questions. A total of 12 supplementary models are reported, each of which augments the core model with questions applicable to specific patient groups/pathways. When considering predictors of care satisfaction in various patient groups including those on specific care pathways, experience of care administration (Q56) and care coordination (Q54) remained the dominant predictors of care satisfaction. The question on shared decision-making about treatment (Q16) was consistently the third strongest predictor, and in-line with the core model its association with overall satisfaction was substantially weaker than care coordination (Q54) and care administration (Q56). However, some patient group/pathway-specific questions were additionally found to be substantial predictors of overall satisfaction, with OR values that were greater than many of the core questions other than the stronger predictors (care coordination [Q54] and administration [Q56]). Examples include the item on being able to discuss worries/fears as an outpatient (Q41) and on the length of waiting time for clinics and appointments (Q57). The question on having been given a care plan (Q55) was consistently not statistically significant in all models. Two further core-model questions became nonsignificant in some models, likely reflecting a weak association combined with a reduced sample size which increased standard errors.

**Table 3.** Results of Supplementary Models Predicting Overall Satisfaction Including Questions Specific to Certain Patient Groups/Pathways.

Number of Observations	Patient Group/Pathway^c^	Question Number	Question Name	OR^b^	LCI^b^	UCI^b^
49 889	Patients who saw a GP before referral/diagnosis of cancer	1	Number of GP visits before hospital referral (prediagnosis)	1.05	1.01	1.09
57 601	Patients having a diagnostic test in last year	7	Tests results explained	1.18	1.15	1.21
51 632	Patients given the name of a clinical nurse specialist	18	Was it easy to contact a clinical nurse specialist	1.25	1.22	1.29
44 260	Patients in work or education^a^	21	Given information about impact on your day to day activities	1.22	1.18	1.26
36 236	Patients who had an operation in past year	26	Operation outcome explained	1.30	1.26	1.35
26 376	Patients who had an operation or overnight stay in hospital in past year	29	Confidence and trust in doctors	1.32	1.27	1.37
		31	Confidence and trust in nurses	1.19	1.14	1.24
		35	Able to discuss worries/fears	1.27	1.20	1.33
		36	Pain control	1.20	1.15	1.24
		38	Clear written information after discharge	1.08	1.04	1.12
50 000	Patients treated as outpatient or day case in past year	41	As outpatient, able to discuss worries/fears	1.41	1.37	1.45
14 961	Patients having radiotherapy in past year	45	Enough info about whether radiotherapy was working	1.21	1.14	1.29
31 736	Patients having chemotherapy in past year	48	Enough info about whether chemotherapy was working	1.28	1.23	1.33
20 962	Patients whose treatment had finished	51	Enough care/support after treatment	1.25	1.18	1.32
47 189	Patients receiving care from GP during treatment	53	GP/practice nurse did their best	1.06	1.02	1.09
66 764	Patients who had attended clinics/appointments	57	Overall length of time to wait for clinics and appointments	1.35	1.31	1.38

Abbreviations: CI, confidence interval; GP, general practitioner; LCI, lower confidence interval; OR, odds ratio; SD, standard deviation; UCI, upper confidence interval.

^a^ Q21 wording implied this question was applicable mainly to those in work or education, although it had no explicit filter question (substantial item nonresponse was seen).

^b^ Odds ratios and 95% CI correspond to a 1 SD change in question response.

^c^ Each set of questions corresponds to a different supplementary model which also adjusts for core-model questions (as well as patient age, gender, deprivation, and tumor group). Odds ratios for all questions contained in each model are shown in Figure 1 to Figure 12 in the Supplement.

A comparison of the ORs for core questions from the core model and the supplementary analyses is shown in Figure 1 to Figure 12 in the Supplement. Although there were some differences, these were mostly within CIs and when they extended beyond the CIs they were still small. Comparison with models identical to the core models but restricted to the appropriate sample suggests that the changes were mostly due to the adjustment of further questions in the model as opposed to sample restriction.

## Discussion

By performing a secondary analysis of responders to the National Cancer Patient Experience Survey, we find that coordination and administration of care are the strongest predictors of patient satisfaction with cancer care, across a diverse range of patient groups/treatment pathways. In the context of our survey, care administration (*Overall, how would you rate the administration of your care [getting letters at the right time, doctors having the right notes/tests results, etc]*) and care coordination (*Did the different people treating and caring for you [such as GP, hospital doctors, hospital nurses, community nurses] work well together to give you the best possible care*) involve different dimensions of care and different care episodes, often by different providers and in different places. In the United Kingdom, most patients care is delivered through a single-health service (the NHS) but within this service, there are many organizations responsible for different aspects of care. Further, cancer care is particularly characterized by multiple and diverse treatment modalities and follow-up appointments involved. A positive experience of involvement in decision-making about treatment, and the patient’s relatives having all the information needed to help care for them, was also substantially associated with patient satisfaction but to a lesser extent. A range of other aspects of patient experience which are generally deemed of importance (including provision of information to the patient and their primary care physician, access to specialist oncology nursing, and experience of waiting time) seem to be weakly only associated with satisfaction with care. In supplementary analyses, examining specific patient groups or care pathways, care coordination and administration remained the strongest predictors of satisfaction with cancer care.

We are not aware of any studies examining satisfaction with care in the context of national patient survey of cancer patients using a “drivers analysis” approach (whereby the influence of patient characteristics and different aspects of experience are taken into account simultaneously). Similar studies, however, have been performed considering the experience of general health-care users in settings such as primary care in the United Kingdom and secondary care in the United States (7,8). Within United Kingdom primary care (7), a positive experience of interaction with practice receptionists was the second most important driver of overall satisfaction (7). In that study, the experience of care administration was not explicitly examined and it may be that receptionists often support the administration of care and therefore the reported association reflected this. Regarding the experience of patients with cancer, our findings concord with those reported by a review of evidence about the importance of care coordination, and its association with other patient-reported outcomes, including satisfaction with care (17). A cancer patient survey from Denmark indicated that 8 different health-care aspects were independently associated with rating of care. Although no item directly measuring care coordination or administration was included in that survey, an item on shared decision-making was found to be a predictor of overall satisfaction ratings (9). A Dutch study directly asked cancer patients which aspects of experience matter most to them (11). Although some findings were concordant with those of the present study (eg, a marker of care coordination “your doctor consults other doctors or refers you if additional expertise is required” was ranked fourth of 83 items and a marker of care administration “the time between first examination and results was short” was ranked fifth), there were also some discordant findings, for example, regarding the importance of diagnostic timeliness. These comparisons illustrate that different analytical approaches can result in findings that are not fully concordant, and study design needs to be kept in mind in interpreting the findings.

In this study, we consistently found no evidence of an association between overall satisfaction and patient reports of being given a care plan. This may seem surprising, however, only a quarter of patients report having been given a care plan. This is similar to the 22% of patients with long-term conditions (not cancer specific) managed in primary care (18). Moreover, patients are often unaware of what care planning is or what it might involve (19), therefore the lack of association may reflect poor understanding of the question. Alternatively, it might be that effective-care planning is put in place without the use of an explicit care plan document. A recent review of the literature indicates that both these mechanisms (which the authors term either lack of “comprehension” or “lack of awareness”) are often present in cancer patients (20).

Strengths of the present study include the large nationwide sample and its high-response rate. This provided the ability to examine the potential importance of a large number of questions as predictors of satisfaction with care with relative precision. Although the response rate was high, filter questions and item nonresponse meant that the number of patients completing all questions was very low. This was addressed by defining a core set of questions and subsequently examining different patient groups/care pathways separately. Further item nonresponse was addressed using multiple imputation. “Drivers analysis” such as the one used in our study is bound by the range of domains examined and the specific questions included in a questionnaire. Consequently, there may be domains of experience not included, or specific questions which are important but not asked. For example, the issue of confidence and trust in those providing care is only addressed to patients staying overnight in hospital. Nonetheless, the Cancer Patient Experience Survey instrument has been constructed with input from cancer patients and advocates; questions encompass the entire journey from diagnosis to outpatient and community care, including items on all key aspects of patient experience (21).

In this study, we concentrated on the drivers of satisfaction comparing those with high scores to those with moderate and low scores. It would have been possible to address the drivers of poor experiences of care focusing on the very lowest scores. However, the less than satisfied group (scoring 7 or less on overall satisfaction) comprised only 14% of responders and thus these are patients with experiences well outside of the norm. We also note that patients often report better experiences than an external observer would, suggesting that scores of 7 or less often represent poor experiences (22,23). Additionally, the use of a lower cutoff point than the one we use would result in a smaller less than satisfied group reducing the power substantially.

The findings can help clinicians and managers engaged in efforts to improve cancer care for patients by principally focusing on improving care coordination (10). Two principal approaches can be considered. First, remodeling care protocols and referral systems to improve informational integration and care co-ordination. This may include the adoption (where not already in place) of integrated electronic patient records, which can be accessed by different care providers. Integrated electronic health records provide promise, nonetheless shared provision of information can also lead to lack of coordination if professional roles and responsibilities between different care providers and specialties are not clearly defined (24). Another approach is to improve “patient navigation” through the employment of specialist staff or volunteers; however, most of the evidence about such care models relates to screening services (25–27).

Further, shared decision-making is an important determinant of care satisfaction; interventions to improve experience of shared decision-making may include longer or “time-out” consultations with treating clinicians, provision of information, or peer-support. Such interventions may principally target patient groups at higher risk of reporting poorer experience of shared decision-making, including younger and ethnic minority patients, and those with cancers of relatively worse prognosis.

Although we have identified domains of care which are strongly associated with overall satisfaction, it is important not to dismiss other aspects of care as unimportant. It may be that the care associated with these aspects of patient experience is universally applied to the same standard across all patients, or that deficits in a given aspect of experience are compensated for by another. While all aspects of patient care and experience should be considered, our findings suggest that it is more important that the different parts of the system work well together. If one part of the system fails to interact with another, then it is not hard to see how there can be a failure in the coordination of care leading to a major impact, much like of a single cog failing can break a whole machine.

## Supplemental Material

Supplemental Material, AbelSupplementJPE - Patient Experience Drivers of Overall Satisfaction With Care in Cancer Patients: Evidence From Responders to the English Cancer Patient Experience Survey Click here for additional data file. Supplemental Material, AbelSupplementJPE for Patient Experience Drivers of Overall Satisfaction With Care in Cancer Patients: Evidence From Responders to the English Cancer Patient Experience Survey by Mayam Gomez-Cano, Georgios Lyratzopoulos and Gary A Abel in Journal of Patient Experience
