# Supplementary material for: Patient Experience Drivers of Overall Satisfaction With Care in Cancer Patients: Evidence From Responders to the English Cancer Patient Experience Survey
Source: J Patient Exp. 2019 Nov 25;7(5):758–65. doi: 10.1177/2374373519889435 (PMC7705845; doi:10.1177/2374373519889435)
Supplement: Supplemental Material, AbelSupplementJPE - Patient Experience Drivers of Overall Satisfaction With Care in Cancer Patients: Evidence From Responders to the English Cancer Patient Experience Survey [file AbelSupplementJPE.pdf]

Patient experience drivers of overall satisfaction with care in cancer patients – Supplement

1. Appendix - supplementary analyses

2. Figures

Figure 1. Results of the supplementary analysis for patients who saw a GP before referral/diagnosis of cancer.

Figure 2. Results of the supplementary analysis for patients having a diagnostic test in last year.

Figure 3. Results of the supplementary analysis for patients given the name of a Clinical Nurse Specialist.

Figure 4. Results of the supplementary analysis for patients in work or education.

Figure 5. Results of the supplementary analysis for patients who had an operation in past year.

Figure 6. Results of the supplementary analysis for patients who had an operation or overnight stay in hospital in past year.

Figure 7. Results of the supplementary analysis for patients treated as outpatient or day case in past year.

Figure 8. Results of the supplementary analysis for patients having radiotherapy in past year.

Figure 9. Results of the supplementary analysis for patients having chemotherapy in past year.

Figure 10. Results of the supplementary analysis for patients who treatment had finished.

Figure 11. Results of the supplementary analysis for patients receiving care from GP during treatment.

Figure 12. Results of the supplementary analysis for patients who had attended clinics/appointments.

1. Appendix - supplementary analyses

The following figures are based on the outputs of the models described in the Supplementary Analyses. These aim to examine whether the findings of the main analysis (which were restricted to items of experience pertaining to all cancer patients) also hold true for specific patient groups (e.g. those defined by specific care pathways such as chemotherapy or radiotherapy).

Each of the figures corresponds to a specific patient group/pathway (with the question(s) applicable only to patients within that specific group/pathway appearing at the bottom of the figure, highlighted in blue). Each figure shows the odds ratio and related 95% CIs for rating cancer care as satisfactory versus less than satisfactory for each independent variable (survey question), for three different models. The red dots show the odds ratio corresponding to the model that augments the core model with the highlighted question(s); the blue dots show the odds ratio corresponding to the core model and; the green dots show the odds ratio corresponding to the model identical to the core model but restricted to the specific patient group (as described on the figures' title).

In nearly all cases the odds ratios for each question are very similar and within the confidence intervals of the estimates of the core model. There are three exceptions. In the model applicable to *patients whose treatment had finished* and the model applying to *patients treated as outpatient or day case in past year* there were changes for one or two questions which resulted in estimates from one model outside the confidence intervals of another. However these differences were too small to be considered of practical impact. In the model restricted to *patients who had an operation or overnight stay in hospital in past year* somewhat larger attenuations were seen for four questions, however they were not large enough to change the overall conclusions.

Comparison of the three sets of models presented here allows an examination of whether any resulting differences in the effect size of "core" questions between the main and supplementary analyses were due to confounding/mediation or sample restriction of the pathway-specific models. In other words, we aimed to distinguish whether aspects of care only experienced by a sub-group of patients on a specific pathway altered the strength of associations between overall satisfaction and common aspects of experience (e.g. through a related mechanism) or whether these patients were inherently different in terms of what mattered to them. These comparisons suggest that the changes were mostly due to the adjustment of further questions in the model as opposed to sample restriction.

2. Figures

Red: The core model and added question(s)
Blue: The core model
Green: The core model excluding missing observations for the added question(s)
In each of the figures, the additional questions (defining specific patient groups / pathways) included in the core model appear in the bottom of the figure, highlighted in blue.

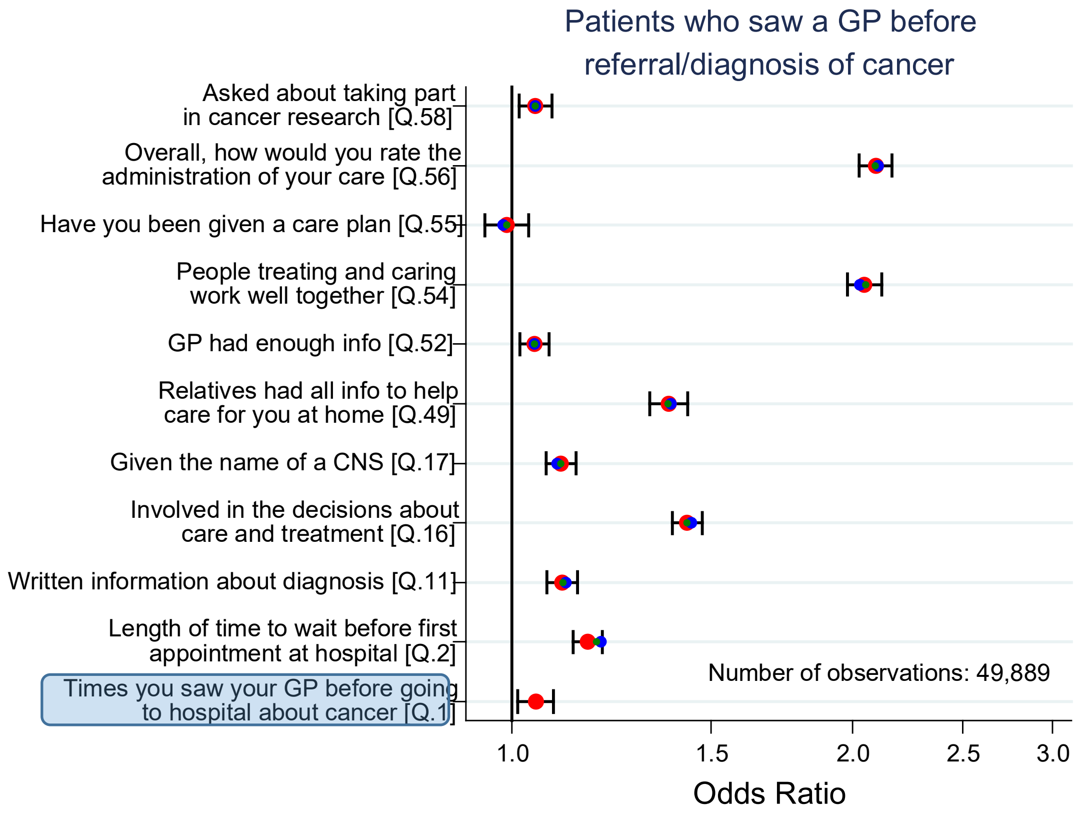

Figure 1. Results of the supplementary analysis for patients who saw a GP before referral/diagnosis of cancer.

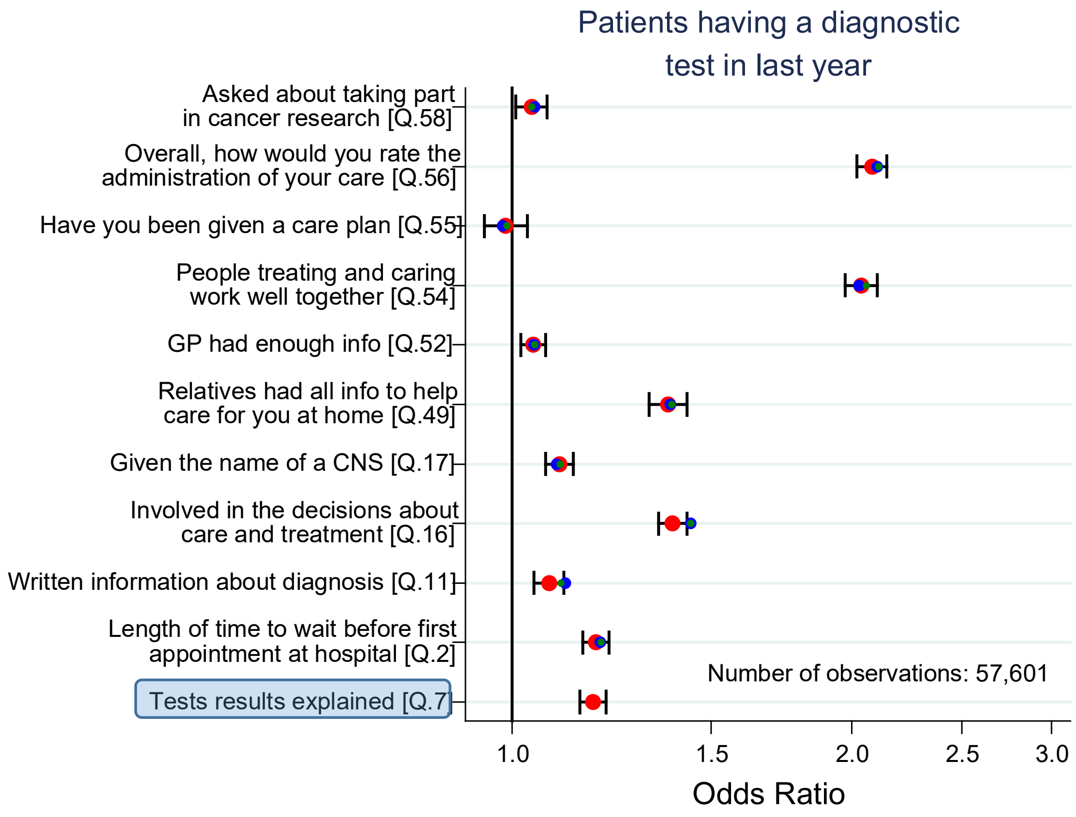

Figure 2. Results of the supplementary analysis for patients having a diagnostic test in last year.

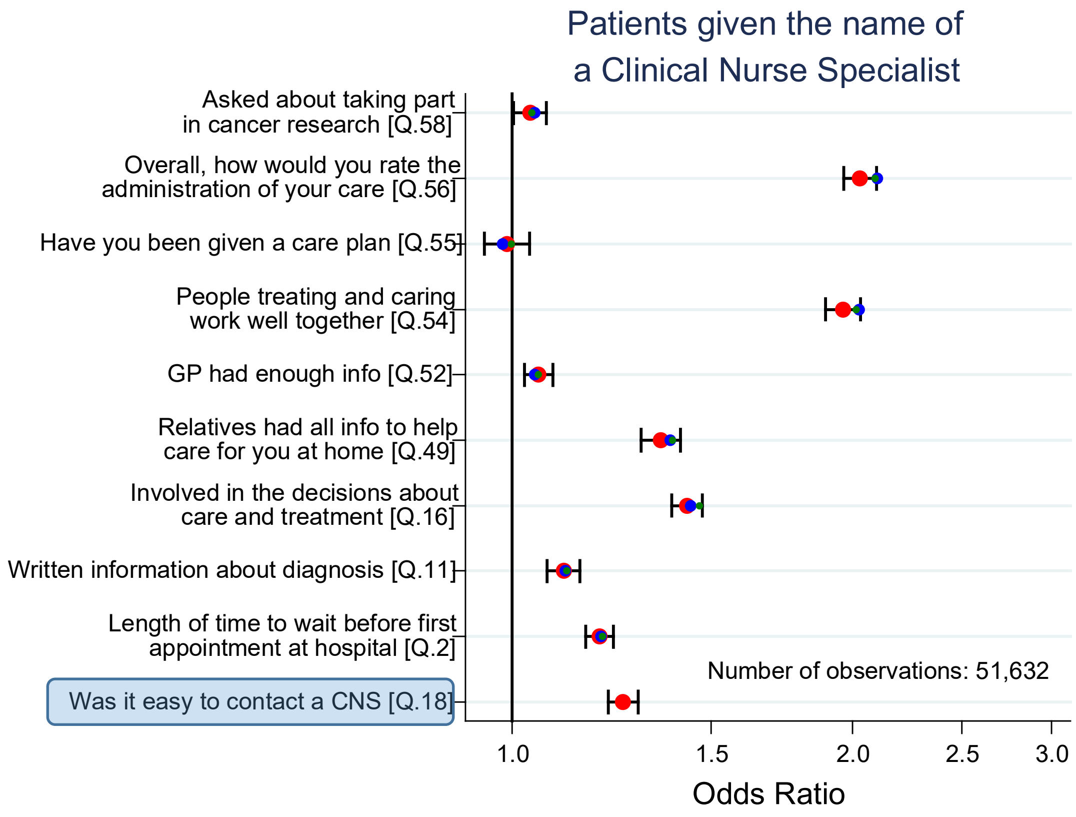

Figure 3. Results of the supplementary analysis for patients given the name of a Clinical Nurse Specialist.

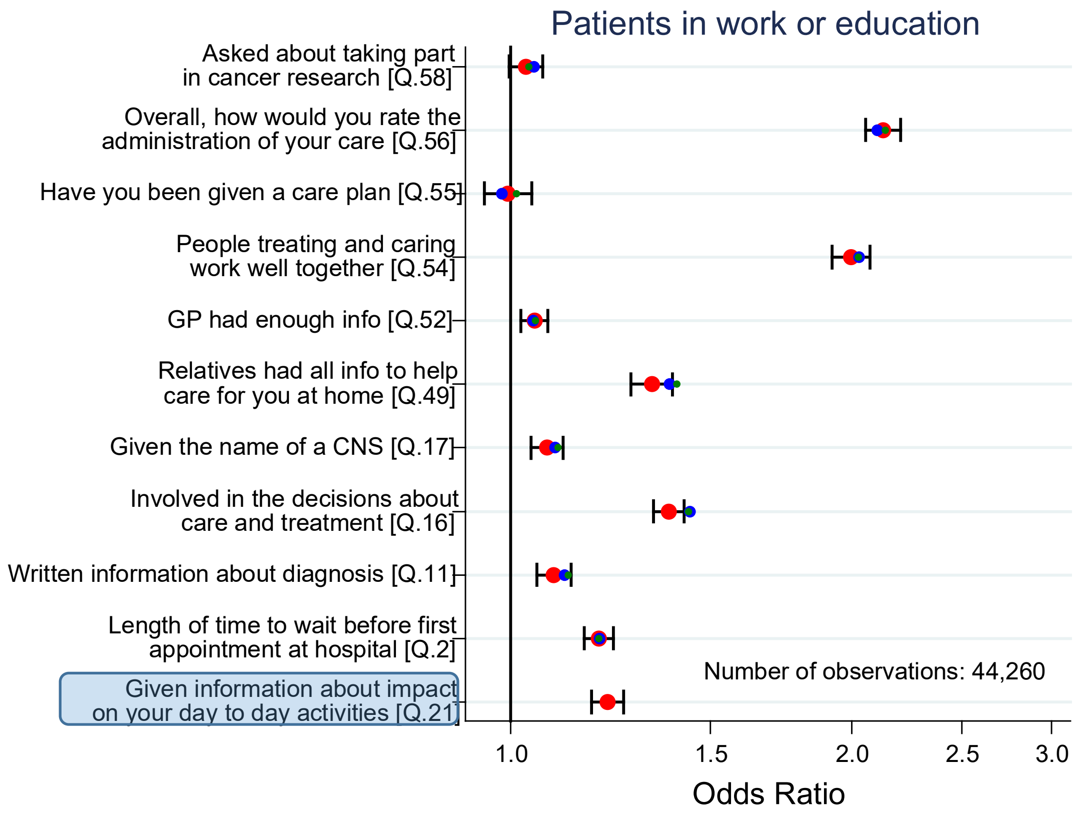

Figure 4. Results of the supplementary analysis for patients in work or education.

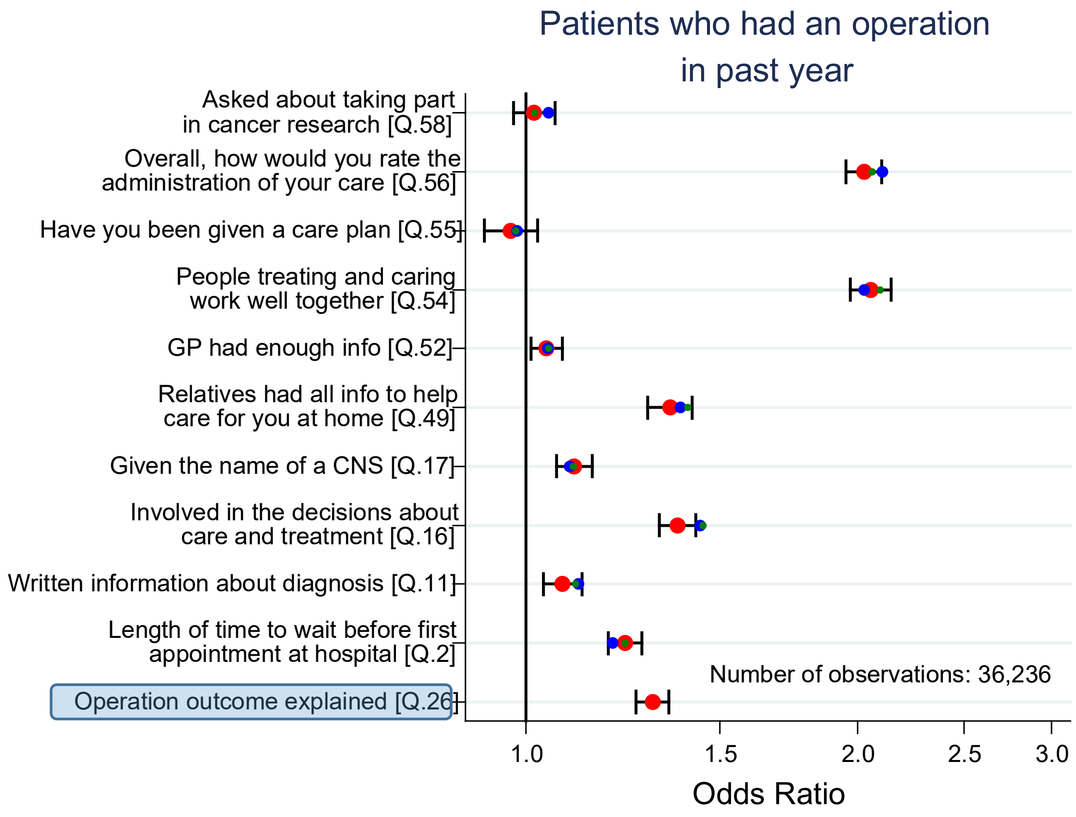

Figure 5. Results of the supplementary analysis for patients who had an operation in past year.

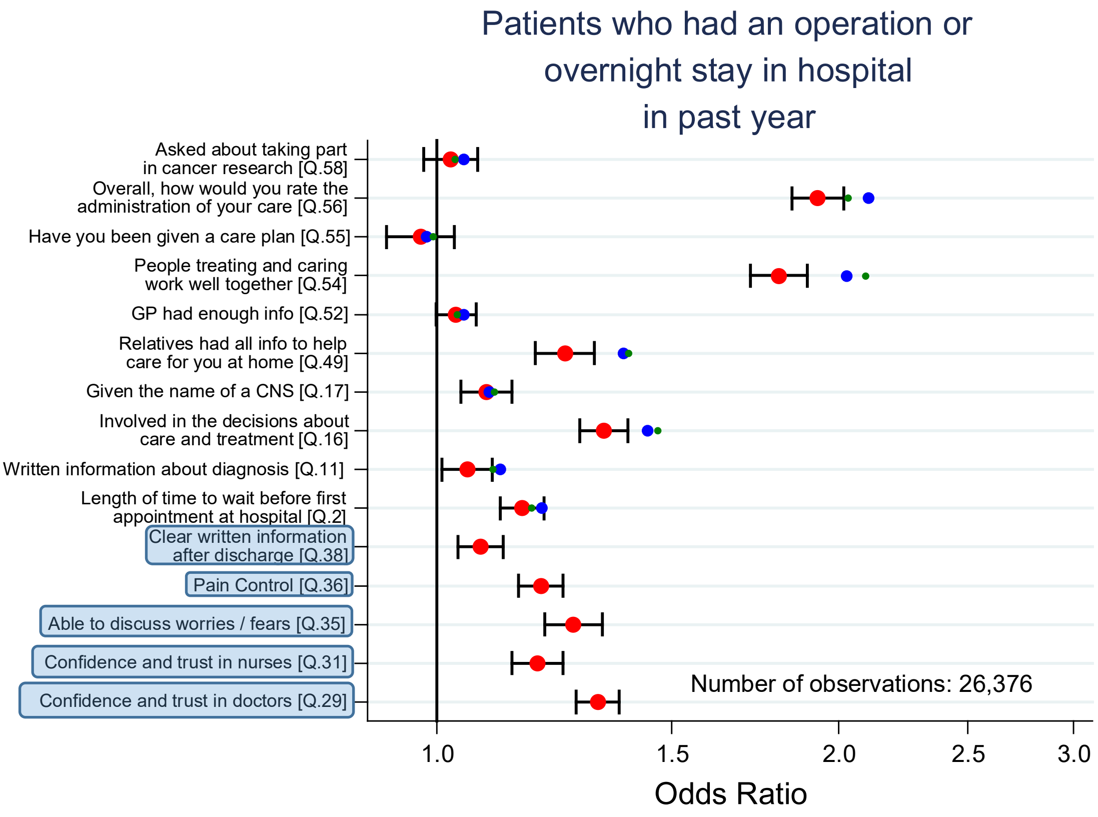

Figure 6. Patients who had an operation or overnight stay in hospital in past year.

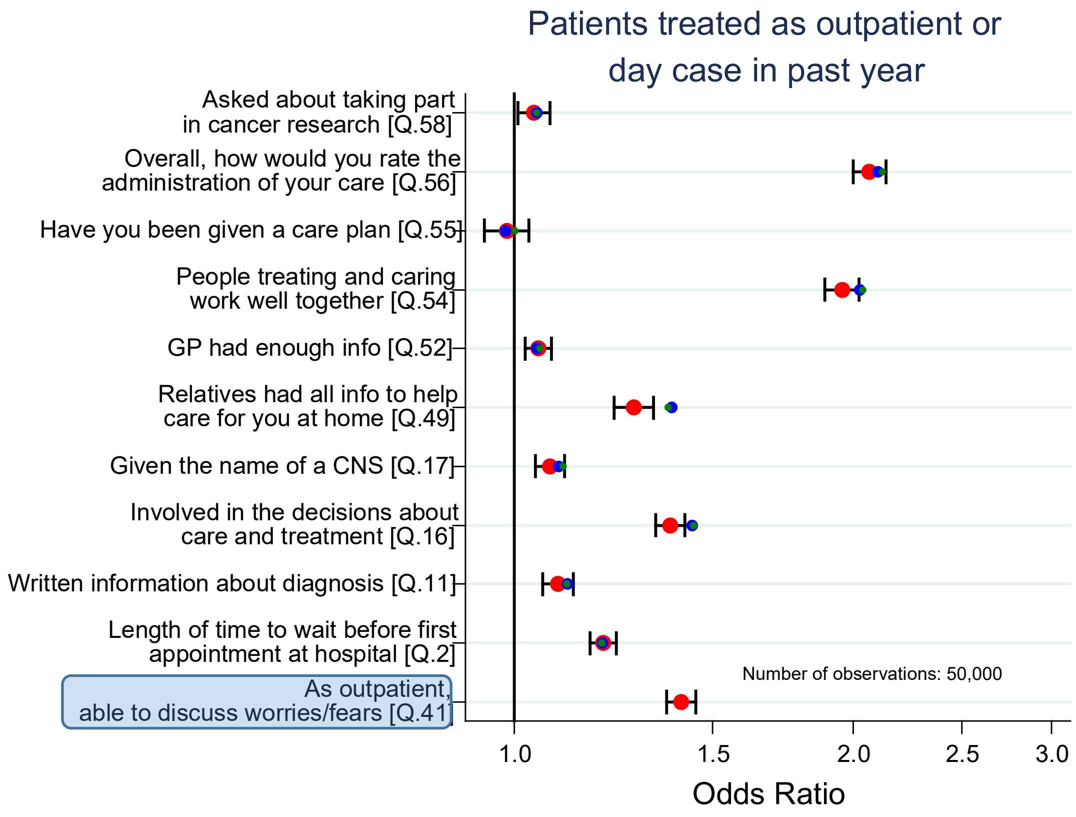

Figure 7. Results of the supplementary analysis for patients treated as outpatient or day case in past year.

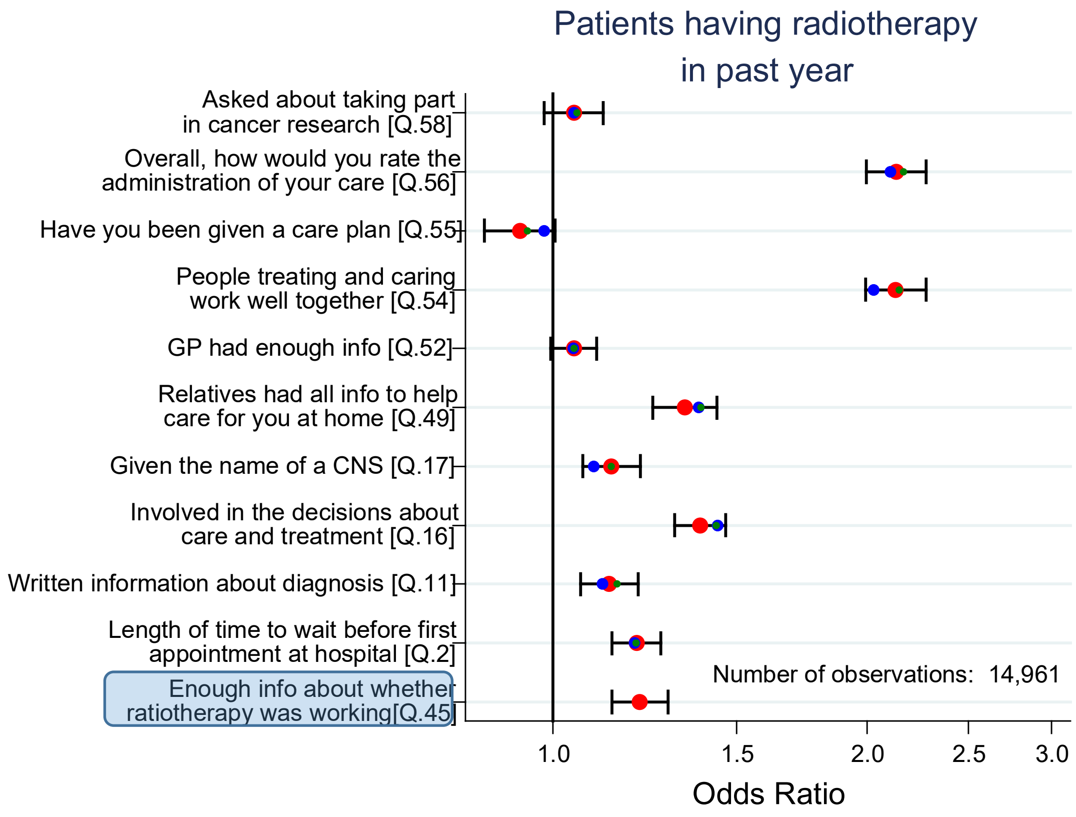

Figure 8. Results of the supplementary analysis for patients having radiotherapy in past year.

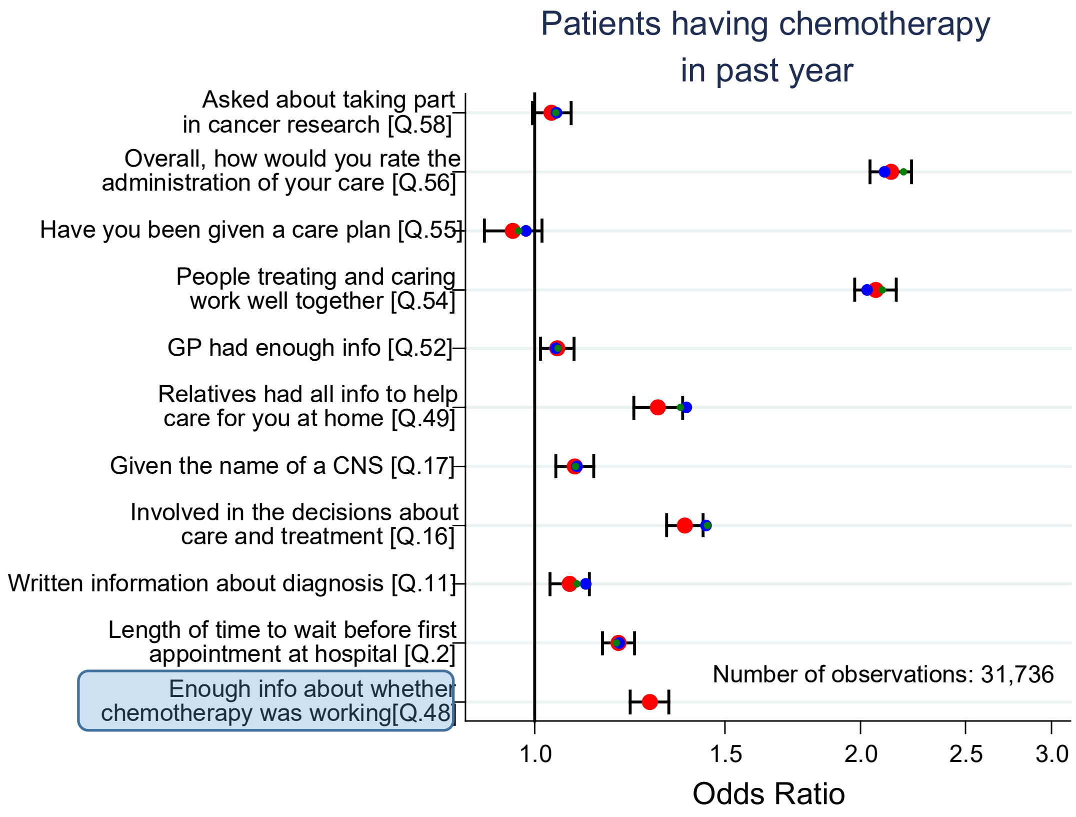

Figure 9. Results of the supplementary analysis for patients having chemotherapy in past year.

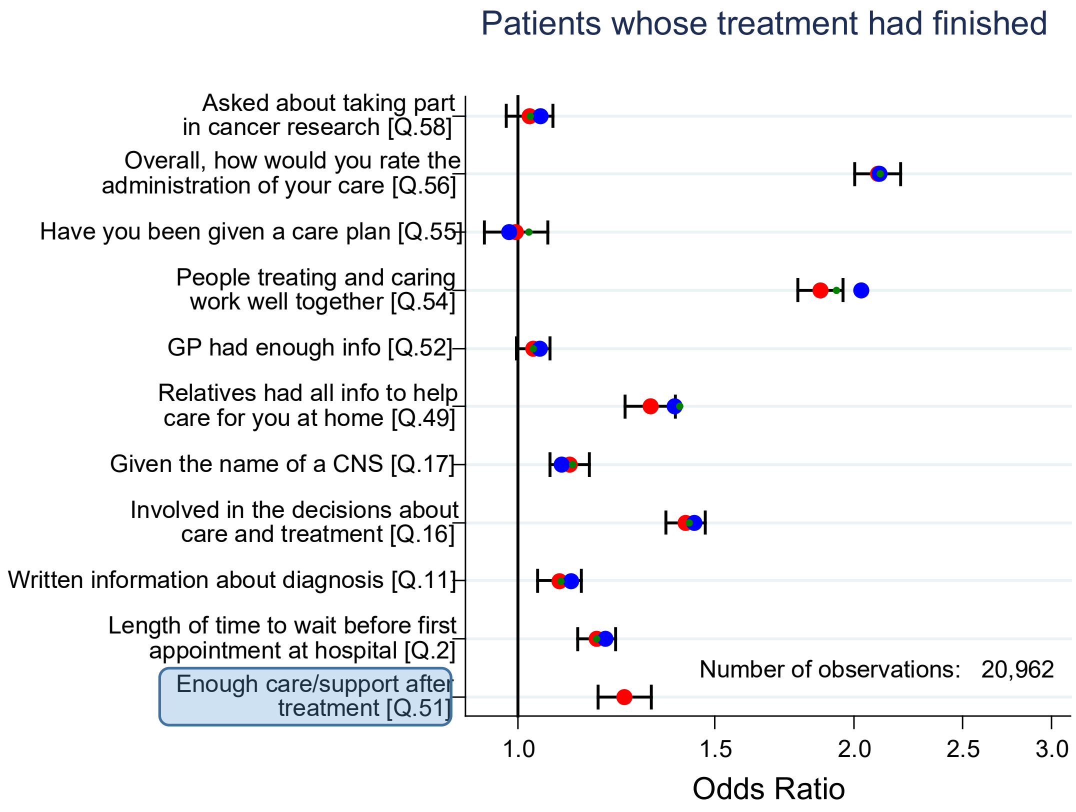

Figure 10. Results of the supplementary analysis for patients whose treatment had finished.

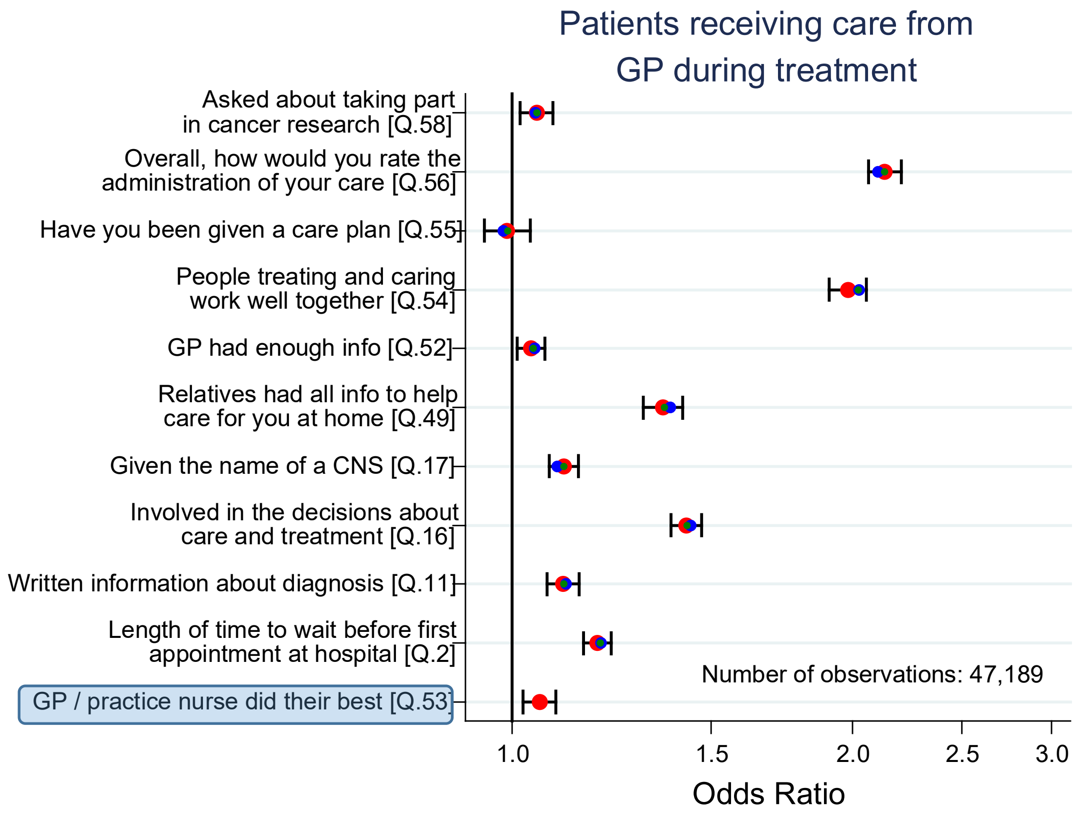

Figure 11. Results of the supplementary analysis for patients receiving care from GP during treatment.

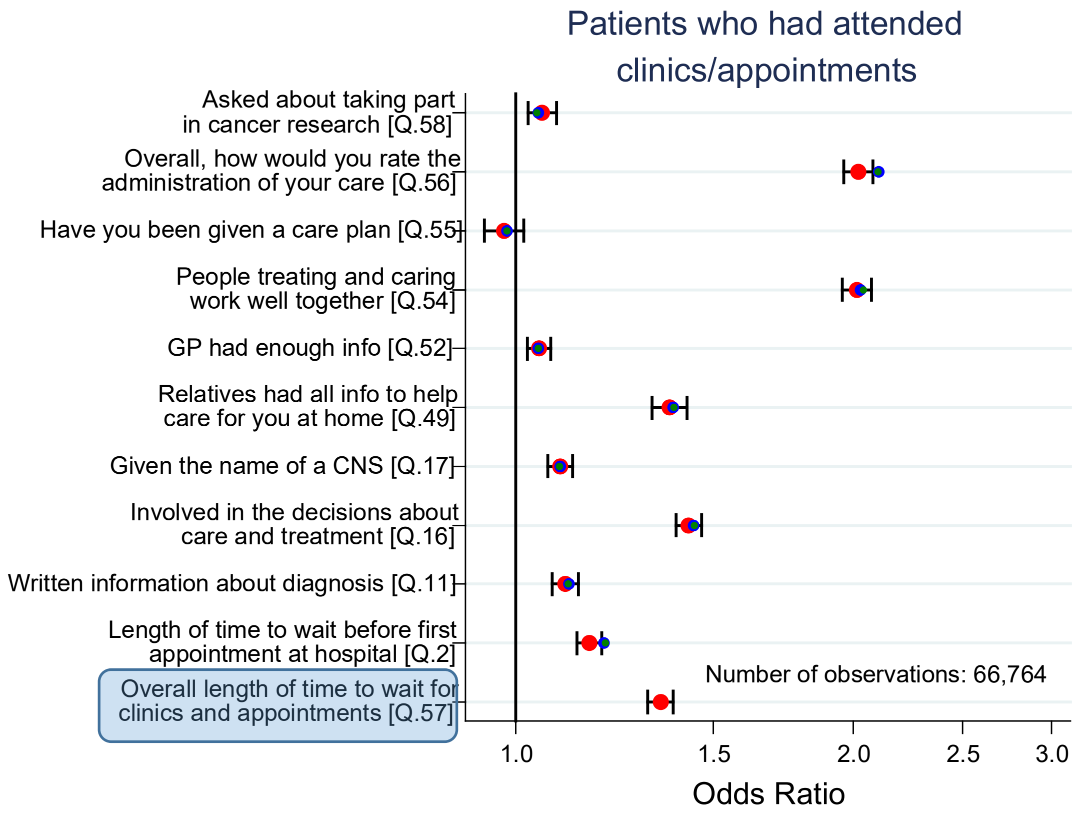

Figure 12. Results of the supplementary analysis for patients who had attended clinics/appointments.
